# Gap-free chromosome-level genomes of male and female spotted longbarbel catfish *Hemibagrus guttatus*

**DOI:** 10.1038/s41597-024-03424-8

**Published:** 2024-06-04

**Authors:** Yexin Yang, Yi Liu, Fangcan Chen, Yuanyuan Wang, Yuli Wu, Zhichao He, Chao Liu, Zhiyong Jiang, Xidong Mu, Chao Bian

**Affiliations:** 1 Key Laboratory of Prevention and Control for Aquatic Invasive Alien Species, Ministry of Agriculture and Rural Affairs, Guangdong Modern Recreational Fisheries Engineering Technology Center, Key Laboratory of Aquatic Animal Immune Technology of Guangdong Province, Pearl River Fisheries Research Institute, Chinese Academy of Fishery Sciences, Guangzhou, China; 2 Laboratory of Aquatic Genomics, College of Life Sciences and Oceanography, Shenzhen University, Shenzhen, 518057 China; 3 Guangdong Hanyu Ecological Technology Co., LTD, Guangzhou, China; 4 Agro-Tech Extension Center of Guangdong Province, Guangzhou, China

**Keywords:** Genome, Data publication and archiving

## Abstract

*Hemibagrus guttatus*, also named as spotted longbarbel catfish, is an economical fish in China. However, their gender cannot be easily distinguished from their appearance, which largely impedes their artificial breeding. Therefore, we provided two gap-free chromosome-level genomes of male and female spotted longbarbel catfish by combining wtdbg2, LR_Gapcloser and TGS-GapCloser assembly approaches with Hi-C data and accurate Pacbio HiFi long-reads. We assembled 30 chromosomes without any gap. Their genome sizes are approximately 749.1 Mb and 747.8 Mb of male and female individuals. The completeness results of BUSCO evaluation show about 94.2% and 95.0%, representing a high-level of completeness of both genomes. We also obtained 35,277 and 34,571 protein-coding gene sets from male and female individuals. Both available gap-free chromosome-level genomes of *H. guttatus* will provide excellent references for resequencing of male and female individuals to identify accurate markers for distinguishing gender of this fish.

## Background & Summary

*Hemibagrus guttatus* (Lacepède, 1803), commonly known as Spotted longbarbel catfish or sesame sword, belongs to the family Bagridae. It has no fish scales and no muscle prickles, inhabiting the bottom of rivers^1^. It was mainly distributed in Pearl River, Yuan Jiang River, Jiulong Jiang River, Han Jiang River, and Qiantang Jiang River in southeastern China, and in Nam Xam and Nam Ma basins of Laos, Red River basin of Viet Nam^1,2^. It is one of “four famous fishes” in Pearl River due to its delicate and tender taste, especially, no fishy taste. In nature, they feed on various foods including crustaceans, insects, fish, annelids, and plant debris, etc^3^.

Due to overfishing and dam construction, the population of *H. guttatus* has sharply decreased in southern China and Viet Nam. Therefore, it was assessed from the IUCN Red List of “Population Decreasing” in 2012^4^. In China, the wild population of *H. guttatus* has been assigned as the National Key Protected Wildlife (the second level) since 2021^5^. In South China, the haplotype diversity and nucleotide diversity of wild *H. guttatus* were relatively low by concatenated COI and Cyt *b* mitochondrial markers^6^. The research results revealed that the population of wild *H. guttatus* is highly homogeneous, and *H. guttatus* may not have had any population expansion in history^6,7^. In Northern Vietnam, no significant differences were found among three wild populations and a farmed population of *H. guttatus* identified by microsatellite markers^8^.

As an economical fish species, *H. guttatus* is in high demand. However, the overfishing has caused serious damage to wild populations, stuck in a vicious circle. Therefore, the key is to accelerate artificial breeding, which will not only protect this species but also meet the supply-demand balance. Many studies focused on biological characteristics and artificial breeding technology of *H. guttatus* have been reported^9,10^. Although there are a few successful cases of reproduction, the low fertilization rate and low survival rate make it impossible to achieve large-scale artificial propagation and breeding. To date, the complete mitochondrial genome sequence of *H. guttatus* has been reported^11^. However, the whole genome has not been reported yet, which is important to understand its genetic diversity and adaptive mechanism, and to improve the artificial breeding efficiency. It is of great significance to obtain the high-quality genome for analyzing the sex determination mechanism and environmental adaptability in fish^12–14^.

Our study reported the first gap-free chromosome-level genomes of both male and female *H. guttatus*. These two genomes also have extremely high ratio of BUSCO (94.2% and 95.0% of male and female genomes). These high-quality and gap-free genomes will be excellent references for the large-scale genome resequencing of both genders in future to discover the correct sex-related makers and then improve the captive breeding efficiency of *H. guttatus*.

## Methods

### Sample collection and DNA extraction

**Fig. 1:**
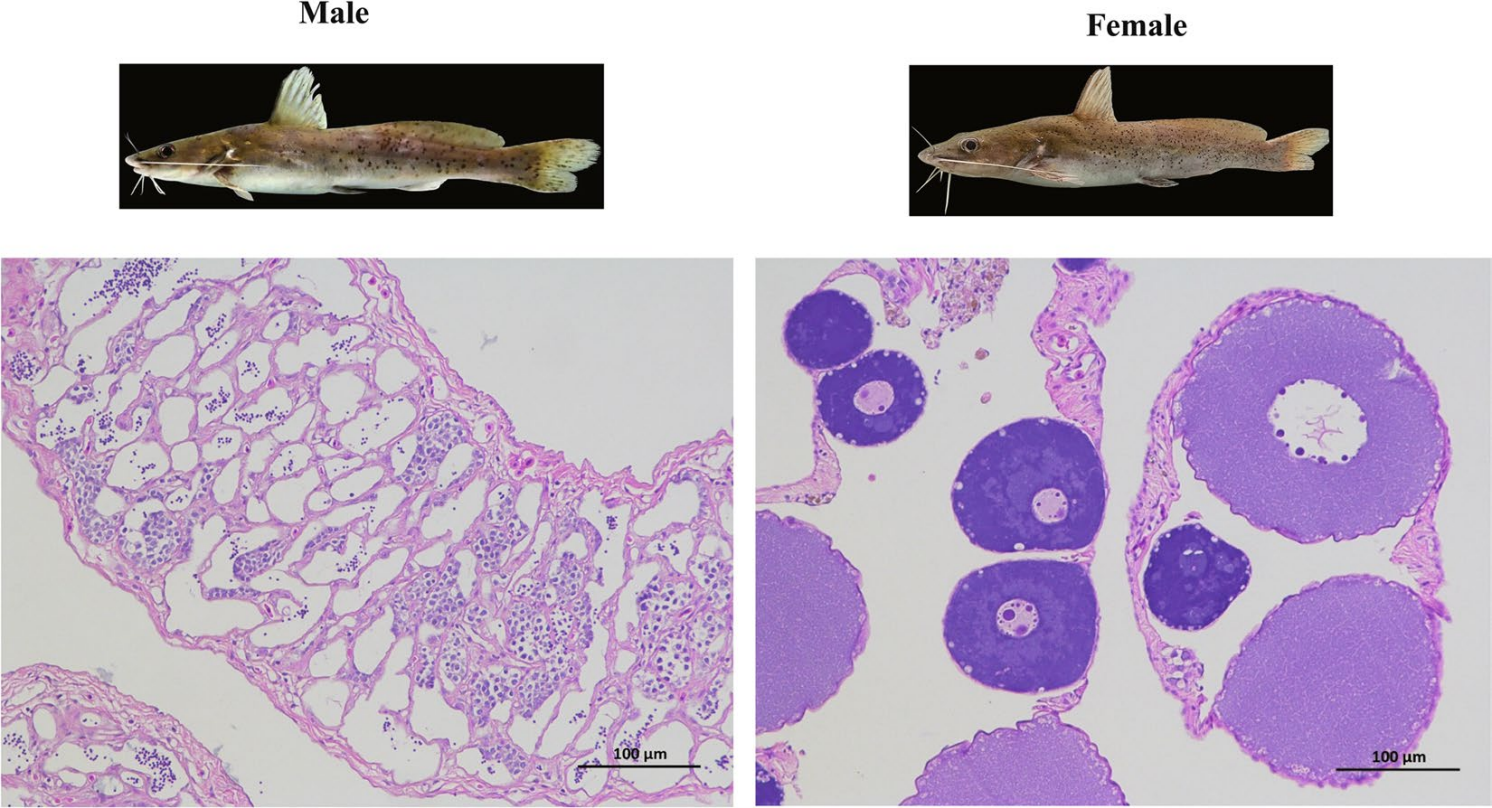
Images of male and female individuals and histological morphology of testis and ovary.

One male and one female of *H. guttatus* were collected from Guangdong Hanyu Ecological Technology Co., LTD, Guangzhou city, Guangdong Province, China (113°30′45″N, 22°55′54″E). The total body lengths of the female and male samples are 36.5 cm and 31 cm, and the body weight of the male and female individuals are 355 g and 670 g. The sample genders were identified by observing gonadal tissue after dissection. Ovary and testis tissues were dissected from samples, fixed by Bouin’s fixative, and embedded in paraffin. The paraffin blocks were cut into 5 μm slices, and were stained with hematoxylin and eosin dye. The stained sections were taken photos by using an Nikon Eclipse Ti-E microscope (Tokyo, Japan, Fig. 1). Fresh muscle tissue was removed below the dorsal fin, freezed in liquid nitrogen quickly, and then stored in a refrigerator at −80 °C in National Freshwater Genetic Resource Center in Guangzhou (http://rc.gibbse.com/#/fontend/navigation). The genomic DNA was extracted according to the instructions of TIANamp Genomic DNA Kit (TIANGEN, Beijing, China). The size of DNA was detected by 1% agarose gel and purity by Qubit 2.0 fluorometer (Life Technologies, USA).

This study protocol was approved by Laboratory Animal Ethics Committee of Pearl River Fisheries Research Institute with licence code: LAEC-PRFRI-2023-02-01.

### Library construction of PacBio HiFi, Hi-C and RNA, and sequencing

Long-read libraries were built with a SMRTbell Express Template Prep Kit 2.0 for PacBio HiFi sequencing by the PacBio Sequel II System according to the PacBio’s standard protocol (Pacific Biosciences, USA). All consensus sequences were produced by using the CCS software (SMRT Link v9.0)^15^. Approximately 19.1 Gb and 21.1 Gb PacBio HiFi reads with average sizes of 17.1 kb and 16.3 kb of male and female individuals were generated.

For Hi-C library construction and sequencing, GrandOmics Hi-C kit and DpnII enzyme (GrandOmics, China) with standard manufacturer protocol were employed to build the Hi-C libraries. The Illumina NovaSeq platform (Illumina, USA) was utilized to perform sequencing of these Hi-C libraries of male and female individuals. A total of 110.3 Gb and 157.4 Gb of Hi-C paired-end reads with 150 bp length were generated for anchoring chromosomes of male and female individuals.

Total RNAs were extracted from the multiple tissues, including heart, liver, gill, muscle and gonad, of female and male individuals by using a TRIZOL Kit (Invitrogen, Carlsbad, CA, USA) with the manufacturer’s instructions. The Agilent 2100 Bioanalyzer System (Agilent Technologies, Santa Clara, CA, USA) was utilized to evaluate purified RNA integrity and quality. The RNAs with RIN (RNA integrity number) >7.0 were selected for library construction. Both male and female cDNA libraries with insert sizes of 300–400 bp were generated in DNA nanoballs (DNBs) according to the manufacturer’s protocol of DNBSEQ sequencing platform. They were then sequenced on a MGISEQ-2000 platform (MGI, BGI Shenzhen, China) to obtain 150-bp paired-end reads.

### Assembling of gap-free chromosome-level genomes and BUSCO evaluation

For primary contig assembly, the sequenced HiFi reads of male and female samples were initially assembled by wtdbg2 software^16^ with detailed parameters (-x ccs -g 900 m -t 32). This step yielded draft assemblies of male and female samples with total sizes of about 749.1 Mb and 747.8 Mb. The detailed contig N50 values male and female samples were 1.3 Mb and 1.4 Mb.

**Fig. 2:**
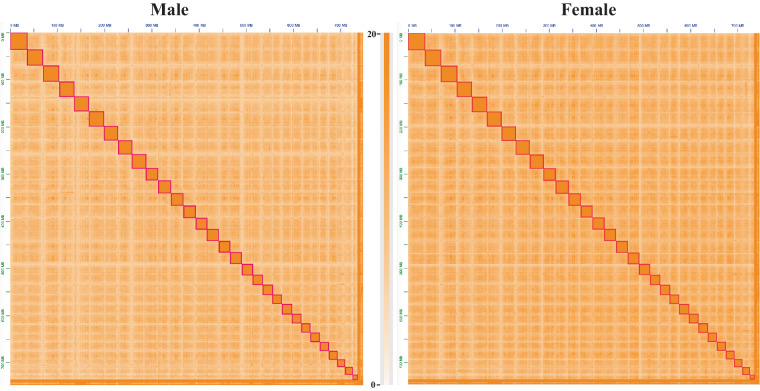
Chromosome heatmaps of Hi-C data of male and female assemblies.

The sequenced Hi-C reads were aligned onto above assembled contigs of male and female samples by employing Bowtie2^17^ (parameters:–very-sensitive -L 30–score-min L, −0.6, −0.2–end-to-end –reorder). Whole valid pair information of chromosome linkage was calculated by HiC-Pro v2.8.0^18^ with default parameters based on the alignment results. This information was then used to anchor contigs into primary chromosomes by using Juicer v1.5^19^ (parameter: chr_num 30) and 3d-DNA v170123^20^ (parameters: -m haploid -r 2)is. The Juicebox v1.11.08^21^ was utilized to correct error-joins, delete duplicated contigs and generate the primary chromosome-level genome assemblies of male and female samples. After generating above chromosome assemblies, male and female chromosome-level assemblies contain 1,706 and 1,612 gaps. We first used the LR_Gapcloser v1.0^22^ (parameters: -t 35 -m 1000000 -v 10000) to fill these gaps of both genomes. In this step, the gap numbers drop down to 3 and 2. The TGS-GapCloser v1.0.1 (parameter:–min_match 2000)^23^ were then performed to fill the remaining gaps of both above assemblies. Finally, 30 gap-free chromosomes with a total length of 737.3 Mb and 737.4 Mb of male and female samples (Fig. 2, Table 1, 2), accounting for 98.4% and 98.6% of the contig-level genome assemblies.

**Table 1:** Summary of gap-free 30 chromosomes of male individual of *H. guttatus*.

Male							
Chr ID	Chr Length	N%	GC%	Chr ID	Chr Length	N%	GC%
Chr01	36,049,790	0	39.56	Chr16	24,460,714	0	39.96
Chr02	34,301,882	0	39.68	Chr17	24,221,947	0	39.96
Chr03	33,835,300	0	39.57	Chr18	23,034,940	0	39.96
Chr04	32,018,624	0	39.95	Chr19	21,346,263	0	39.86
Chr05	31,731,893	0	39.86	Chr20	20,928,263	0	39.78
Chr06	31,674,479	0	39.55	Chr21	20,538,562	0	39.95
Chr07	30,285,971	0	39.86	Chr22	20,429,466	0	39.97
Chr08	29,316,885	0	39.74	Chr23	20,243,556	0	39.76
Chr09	29,274,011	0	39.58	Chr24	19,625,622	0	39.99
Chr10	26,640,510	0	39.54	Chr25	19,285,685	0	40.11
Chr11	26,313,708	0	39.72	Chr26	18,960,754	0	40.08
Chr12	26,152,176	0	39.68	Chr27	18,065,623	0	40.17
Chr13	25,904,549	0	39.85	Chr28	16,375,464	0	40.55
Chr14	24,689,583	0	39.82	Chr29	16,274,039	0	40.36
Chr15	24,608,520	0	39.97	Chr30	10,721,353	0	41.52

**Table 2:** Summary of gap-free 30 chromosomes of female individual of *H. guttatus*.

Female							
Chr ID	Chr Length	N%	GC%	Chr ID	Chr Length	N%	GC%
Chr01	36,275,100	0	39.56	Chr16	24,691,143	0	39.99
Chr02	34,270,156	0	39.63	Chr17	24,240,819	0	39.94
Chr03	33,829,289	0	39.53	Chr18	22,807,985	0	39.88
Chr04	32,258,151	0	39.92	Chr19	21,496,477	0	39.84
Chr05	32,107,587	0	39.55	Chr20	20,784,492	0	39.73
Chr06	31,149,686	0	39.74	Chr21	20,759,521	0	39.94
Chr07	30,018,353	0	39.84	Chr22	20,383,845	0	39.77
Chr08	29,368,011	0	39.56	Chr23	20,169,949	0	39.99
Chr09	29,165,018	0	39.69	Chr24	20,032,235	0	40.02
Chr10	26,538,388	0	39.54	Chr25	19,269,869	0	40.06
Chr11	26,349,448	0	39.73	Chr26	18,913,609	0	40.01
Chr12	26,234,021	0	39.7	Chr27	18,296,424	0	40.17
Chr13	25,691,565	0	39.81	Chr28	16,696,201	0	40.47
Chr14	24,767,560	0	39.8	Chr29	15,696,575	0	40.24
Chr15	24,740,407	0	39.91	Chr30	10,423,826	0	41.33

### Repeat annotation

The homology and *de novo* prediction pipelines were performed to identify repeat elements in above anchored chromosome-level genomes. In *de novo* prediction, RepeatModeler v1.0.8^24^ and LTR-FINDER v1.0.6^25^ were utilized to identify diverse types of repetitive sequences. A novel library was created using RepeatMasker v4.0.623^26^ on the basis of the Repbase TE v21.01^27^. Tandem repeat sequences were found employing the Tandem Repeats Finder^28^ with detailed parameters: 2 7 7 80 10 50 2000 -d -h. Using above repeat library from the *de novo* prediction, RepeatProteinMask v4.0.623^26^ and RepeatMasker v4.0.623^26^ with default parameters were utilized to look for repeat sequences in both genome assemblies. The centromere sequences were identified by the quarTeT software^29^.

**Fig. 3:**
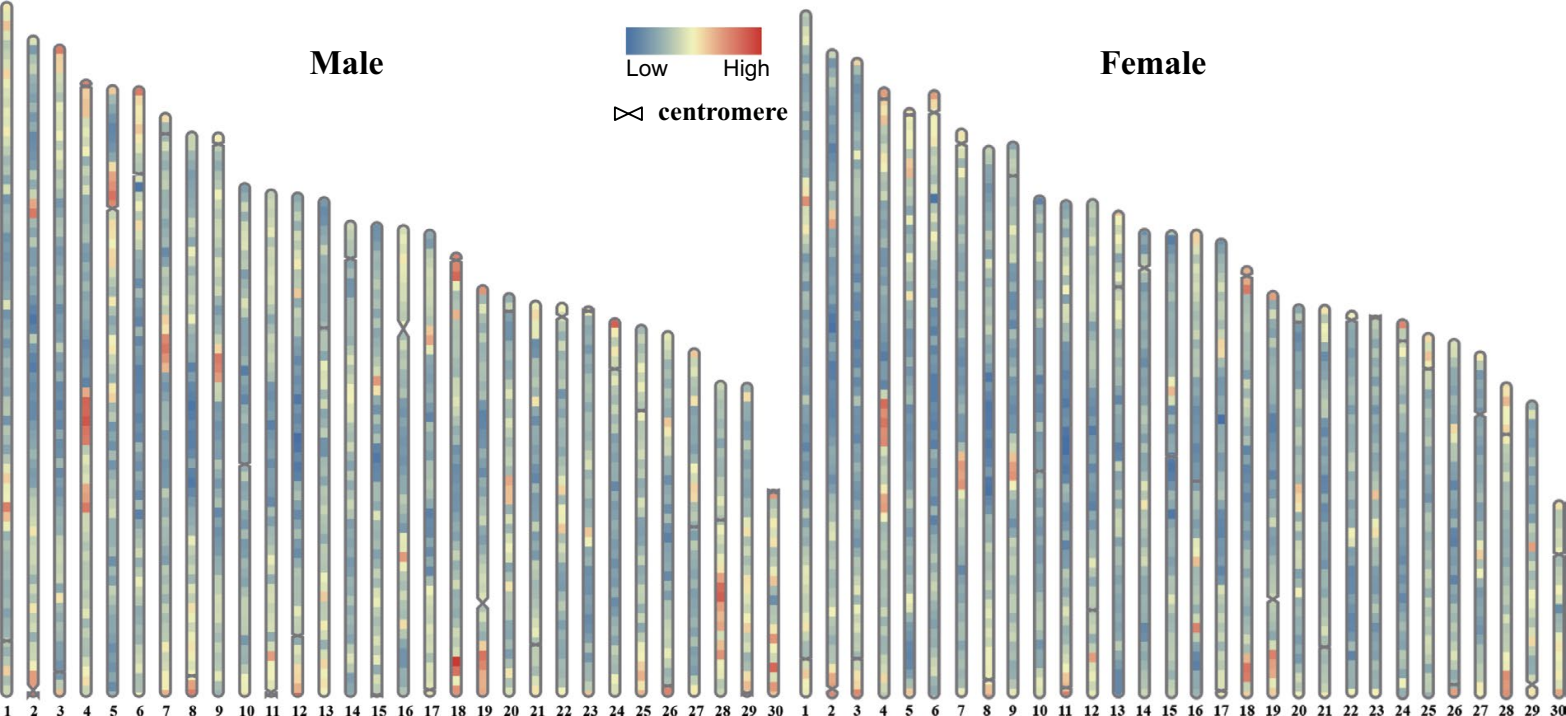
Repeat distribution and centrosome locations in male and female assemblies.

Finally, we estimated that male and female genomes contain roughly 312.3 Mb and 311.7 Mb of repetitive sequences, accounting for about 41.7% and 41.7% of their genomes. The detailed distribution of repeat sequences and centromere were shown in Fig. 3.

### Gene prediction and functional annotation

We integrated two approaches to annotate the structure of protein-coding genes, including homology-based annotation and transcriptome-based annotation, to provide a completely predicted gene set. Protein sequences from five representative teleosts, including *Tetraodon nigroviridis*, *Ameiurus melas*, *Takifugu rubripes*, *Oryzias latipes*, and *Danio rerio*, were downloaded from the NCBI database (release 84) for aligning onto the male and female genome assemblies by TBLASTn^30^ with an e-value 10^−5^ for the homology-based annotation.

On the basis of above-mentioned tBLASTn alignments, the GeneWise v2.2.0^31^ (parameters:–blast_eval 1e-5–align_rate 0.5–extend_len 500) was used to identify gene structures. About 12.4 Gb and 15.1 Gb pooled RNA-seq data of male and female samples were mapped using HISAT^32^ onto the male and female genome assemblies for the transcriptome-based prediction. The RNA-seq alignments were analyzed by using Cufflinks v2.2.1 (http://cole-trapnell-lab.github.io/cufflinks/) for identifying gene structures. To create a final non-redundant gene set, MAKER^33^ with parameters: max_dna_len = 300000, min_contig = 500, pred_flank = 500, AED_threshold = 1, split_hit = 30000, single_exon = 1, single_length = 250, tries = 2 was used to combine all above gene sets from the aforementioned two methods. By matching it against three public databases, including SwissProt^34^, TrEMBL^35^, and KEGG (Kyoto Encyclopedia of Genes and Genomes)^36^, it was functionally annotated. The InterProScan^37^ application was used to annotate the gene ontology (GO).

A total of 35,277 and 34,571 protein-coding genes were estimated in male and female individuals with mean length of 12,652.1 bp and 12,872.0 bp. With mean length of 218.3 bp and 218.6 bp, each gene contains an average of 7.7 and 7.7 exons. The majority of the predicted genes, around 98.6% (34,786 genes) and 98.7% (34,103 genes), were given at least one function (Table 3).

**Table 3:** Statistics of the assembly and annotation results of male and female individuals of *H. guttatus*.

	Male	Female
HiFi reads (Gb)	19.1	21.1
Hi-C reads (Gb)	110.3	157.4
Genome size (Mb)	749.1	747.8
Contig N50 (Mb)	25.9	25.7
BUSCO	94.2%	95.0%
Repeat ratio	41.7%	41.7%
Gene number	35,277	34,571
Average gene length (bp)	12,652.1	12,872.0
Functional gene number	34,786	34,103

## Data Records

Both final genome assemblies, gene sets and raw reads of the male and female individuals are available at NCBI with accession numbers: JAUCMX000000000^38^ and JAUCMY000000000^39^. The annotation coding sequences and protein sequences were deposited at Figshare with doi number 10.6084/m9.figshare.24130344^40^. The raw reads of PacBio and Illumina sequencing were also uploaded at the NCBI with accession numbers SRP459419^41^ and SRP459422^42^.

## Technical Validation

We confirmed that both male and female have 30 chromosomes by using high-quality Hi-C assemblies. The HiFi reads were aligned onto both male and female genome assemblies by using Minimap2. The mapping ratio are 99.97% and 99.98% from male and female assemblies. We also performed the assessment of the completeness of both male and female genome assemblies by using Benchmarking Universal Single-Copy Orthologs (BUSCO) v.5.2.2 with the Actinopterygii reference (3,640 single-copy orthologs; OrthoDB v.10). A total of 94.2% (3,428) and 95.0% (3,456) BUSCOs were identified as complete. Of these, 93.4% (3,398) and 94.1% (3,424) were single-copy, and 0.8% (30) and 0.9% (32) were duplicated. For annotation BUSCO results, a total of 88.6% (3,226) and 89.6% (3,262) BUSCOs were identified as complete. Of these, 87.1% (3,170) and 88.0% (3,202) were single-copy, and 1.5% (56) and 1.6% (60) were duplicated. Taken together, these results confirmed the high quality of both male and female genome assemblies and annotation results.

## Data Availability

All scripts and pipelines used for the genome assembly and gene annotation were performed according to their manuals and protocols of the applied bioinformatics software. No specific code has been developed for this study.
